# Observing animals and humans: dogs target their gaze to the biological information in natural scenes

**DOI:** 10.7717/peerj.10341

**Published:** 2020-12-16

**Authors:** Heini Törnqvist, Sanni Somppi, Miiamaaria V. Kujala, Outi Vainio

**Affiliations:** 1 Department of Equine and Small Animal Medicine, Faculty of Veterinary Medicine, University of Helsinki, Helsinki, Finland; 2 Department of Psychology, Faculty of Education and Psychology, University of Jyväskylä, Jyväskylä, Finland

**Keywords:** Scene, Family dogs, Kennel dogs, Animal images, Human images, Biological information, Cognition, Eye gaze tracking, Full body images

## Abstract

**Background:**

This study examines how dogs observe images of natural scenes containing living creatures (wild animals, dogs and humans) recorded with eye gaze tracking. Because dogs have had limited exposure to wild animals in their lives, we also consider the natural novelty of the wild animal images for the dogs.

**Methods:**

The eye gaze of dogs was recorded while they viewed natural images containing dogs, humans, and wild animals. Three categories of images were used: naturalistic landscape images containing single humans or animals, full body images containing a single human or an animal, and full body images containing a pair of humans or animals. The gazing behavior of two dog populations, family and kennel dogs, were compared.

**Results:**

As a main effect, dogs gazed at living creatures (object areas) longer than the background areas of the images; heads longer than bodies; heads longer than background areas; and bodies longer than background areas. Dogs gazed less at the object areas *vs.* the background in landscape images than in the other image categories. Both dog groups also gazed wild animal heads longer than human or dog heads in the images. When viewing single animal and human images, family dogs focused their gaze very prominently on the head areas, but in images containing a pair of animals or humans, they gazed more at the body than the head areas. In kennel dogs, the difference in gazing times of the head and body areas within single or paired images failed to reach significance.

**Discussion:**

Dogs focused their gaze on living creatures in all image categories, also detecting them in the natural landscape images. Generally, they also gazed at the biologically informative areas of the images, such as the head, which supports the importance of the head/face area for dogs in obtaining social information. The natural novelty of the species represented in the images as well as the image category affected the gazing behavior of dogs. Furthermore, differences in the gazing strategy between family and kennel dogs was obtained, suggesting an influence of different social living environments and life experiences.

## Introduction

Visual attention, often measured as ‘looking preferences’, is commonly used to assess animal behavior and cognition (Winters, Dubuc & Higham, 2015). Eye gaze tracking provides more quantifiable and specific information, and during the past decade, it has been successfully applied when investigating dogs’ responses towards still images. Previously, dogs have been found to spontaneously discriminate human and dog faces from each other and from inanimate objects (Racca et al., 2010; Somppi et al., 2012; Gergely et al., 2019) and even facial expressions of humans and dogs (Nagasawa et al., 2011; Somppi et al., 2016; Somppi et al., 2017). Dogs can learn to differentiate facial images of dogs from the facial images of other animal species (Autier-Dérian et al., 2013) and to discriminate between naturalistic images that contain a dog from those that do not (Range et al., 2008). However, most of the studies using looking-time paradigms have used facial images and unlike primate studies (Kano & Tomonaga, 2009; Kano, Call & Tomonaga, 2012) little canine research has used naturalistic whole-body images of humans and animals (Törnqvist et al., 2015), or different kinds of images (e.g., landscapes and faces) embedded in the same study design (Range et al., 2008; Autier-Dérian et al., 2013). Extending the research of canine gazing behavior to more naturalistic images provides a unique opportunity to study both the effect of image composition and object or category content at the same time.

The capacity to respond appropriately to biologically relevant stimuli (prey, predators and social partners) is crucial for animal survival, and it has been suggested to be an unlearned social predisposition (for reviews, Rosa-Salva, Mayer & Vallortigara, 2015; Di Giorgio et al., 2017). The primate visual system is specialized in detecting living creatures from complex natural scenes (Fabre-Thorpe, Richard & Thorpe, 1998; Thorpe et al., 2001), and typically humans and monkeys focus their gaze specifically on the head area of these creatures (e.g., Yarbus, 1967; Kano & Tomonaga, 2009; Kano, Call & Tomonaga, 2012). Biologically relevant stimuli such as animals attract attention more than inanimate objects and social information draws more attention than non-social (e.g., in non-human primates: Machado et al., 2011; Hanazuka et al., 2012; in humans: New, Cosmides & Tooby, 2007; Simion, Regolin & Bulf, 2008; in dogs: Somppi et al., 2012; Törnqvist et al., 2015). Dogs’ spontaneous preferences toward social information has also been recently demonstrated with biological motion videos (Kovács et al., 2016; Ishikawa et al., 2018).

The amount of visual experience a person has in a particular domain can promote an expert cognitive strategy in processing visual information (e.g., Diamond & Carey, 1986; Kundel et al., 2008; Kujala et al., 2012). For example, when viewing images of two dogs interacting with or facing away from one another, dog experts gaze relatively longer at the dogs’ bodies than at their heads when compared with non-experts (Kujala et al., 2012). Dog owners assess the approachability of dogs by assessing their facial expression more rapidly and with fewer fixations than non-dog owners, but when assessing human and monkey faces, both groups have similar viewing behavior (Gavin, Houghton & Guo, 2017). Also human infants, who have previous experience with pets, focus their attention more on the head areas in cat and dog images than infants without pet experience (Kovack-Lesh, McMurray & Oakes, 2014). When humans and non-human primates observe social videos of other animals demonstrating their species-typical behaviors, the distinct viewing patterns exhibited by these observers are influenced by their previous experiences of that species (Kano et al., 2018). Likewise, the amount of previous exposure also affects dogs’ gazing behavior during the presentation of human and dog faces (Somppi et al., 2014; Barber et al., 2016) and natural images of social interaction (Törnqvist et al., 2015).

In the present study, we measured the gazing behavior of family and kennel dogs while they viewed natural images of dogs, humans and wild exotic animals. Three categories of images were used: landscape images containing single humans or animals; full body images containing a human or an animal; and full body images containing a pair of humans or animals of the same species. We hypothesized that when dogs viewed naturalistic images, dogs would focus more attention on living creatures—heads and bodies of animals and humans—even when the figure was small in relation to the image. In addition, we assumed that the composition of the images would affect dogs’ gazing behavior. We also anticipated that dogs would gaze at the heads and bodies of living creatures, even when viewing unfamiliar species of animals; and that dogs would focus more on their conspecifics than non-conspecifics. Furthermore, we assumed that family and kennel dogs’ social experiences would affect the way in which they viewed biological information.

## Materials & Methods

### Ethical statement

This study was conducted in strict accordance with the Finnish Act of Animal Experimentation (62/2006) and with fully implemented European convention for the protection of vertebrate animals used for experimental and other scientific purposes (Directive 86/609/EEC). The study was approved by the Ethical Committee for the Use of Animals in Experiments at the University of Helsinki (2/2010) and the Finnish national Animal Experiment Board (approval#STH367A/ESLH-2008-04236/Ym-23). Dogs were not harmed in any way and their movements were not restricted during the training or the study. Only non-invasive methods were used during the measurements.

### Subjects

A total of 16 privately owned family dogs and eight kennel dogs participated in the study. Family dogs (11 females and five males) represented nine different breeds and two mongrels. They were 1–8 years old [4.3 ± 2.2 years (mean ± standard deviation)] and lived with their owners. Their daily routine consisted of food provision once or twice a day and being taken outdoors three to five times daily. Family dogs regularly saw dogs and other animal species (cats, birds etc.) in their living environment. They lived in the homes of their owners and constantly interacted with their human families. Family dogs did not have any specific behavioral disturbances (e.g., anxiety or aggression). Kennel dogs (two females and six males) were purpose-bred beagles that lived together as a social group in a kennel-like environment at the facilities of University of Helsinki. During the experiment, the kennel dogs were 4 years old. They were given food twice a day and were taken to an outside exercise area for 2 h once a day. Kennel dogs did not meet dogs of other breeds or any other animal species, but they regularly saw other beagle dogs living in the same building. They were rarely taken outside the kennel and seldom met other humans except the caretakers and the researchers with whom they were familiar. Compared to family dogs, kennel dogs were quite fearful and cautious in training and experimental situations. All the dogs had participated in eye tracking experiments prior to the present experiment, but they were naïve to stimuli image content.

### Training of the dogs

Before the experiment, dogs were trained to lie still and lean their heads on a U-shaped chin rest during the presentation of the images, as in our previous experiments (e.g., Somppi et al., 2012; Törnqvist et al., 2015). Dogs were trained with the positive operant conditioning method (clicker). Owners trained their family dogs as instructed by the experimenters and kennel dogs were trained by the experimenters. Dogs were neither encouraged to fixate on a screen or images during the training nor trained for visual discrimination tasks. The dogs were not physically restrained, and they took the pre-trained position of their own volition without commands (for more details, see Somppi et al., 2012).

### Eye gaze tracking and calibration

The eye gaze measurements were conducted at the Faculty of Veterinary Medicine at the University of Helsinki. Dogs’ binocular eye movements were measured with an infrared contact-free eye tracker (iView XTM RED250, SensoMotoric Instruments GmbH, Berlin, Germany) integrated under liquid-crystal display (LCD). The data were collected with a sampling rate of 250 Hz. The distance between dog’s eyes and the screen was 0.61–0.73 m, and the distance depended on the size of the dog (smaller dogs were closer to the screen). The screen, eye tracker and the chin rest were placed in a cardboard cabin (height = 1.5 m, width = 0.9 m, depth = 0.9 m) that had three walls and a roof (see Somppi et al., 2012) for a schematic picture of the setup). In front and above the screen were fluorescent lamps, and the intensity of illumination was 4,200–13,400 lx (11,000 ±   2,300 lx) measured on top of the chin rest. Calibration of the eye tracker for each dog’s eyes was done using a five-point procedure (see Somppi et al., 2012). The screen was replaced with a plywood wall with five 30-mm holes in the calibration point positions, and the experimenter lifted up a flap covering the hole and showed a treat in the hole to catch the dog’s attention. Two additional calibration check trials were conducted after the calibration for verification. Calibration were repeated one –13 times (4 ±   0.6) for each dog in order to obtain an optimal calibration. The average calibration accuracy was 95%, which was calculated as a proportion of the fixated points out of five calibration points over two calibration checks. To maintain dogs’ ideal vigilance and to prevent frustration, calibration and the eye gaze measurements were done on separate days. During the calibration, calibration checks and eye gaze measurements the position of the head, illumination and eye tracker position were maintained the same.

### Stimuli

**Figure 1:**
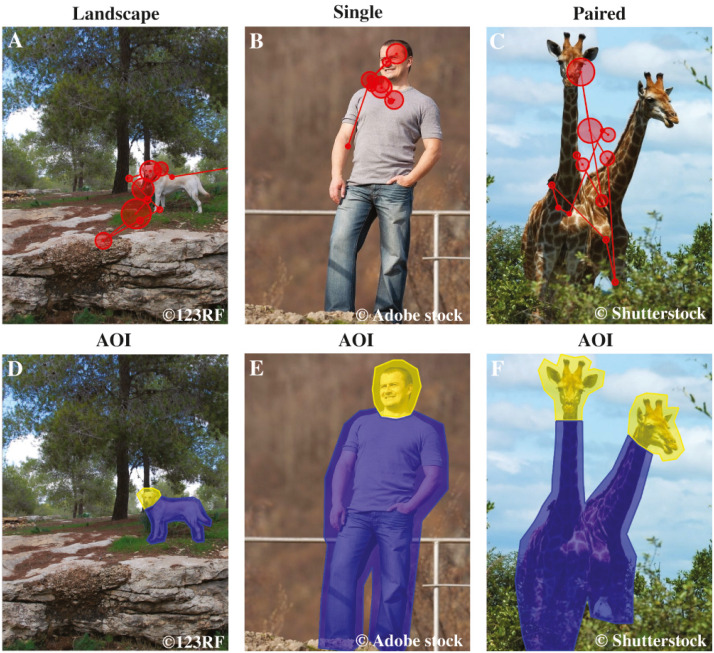
Examples of AOI areas, stimulus images and dog’s scan paths. The first row shows examples of the stimuli of the different image categories: Landscape, Single and Paired, and a dog’s scan paths to the images. Here the example images are (A) landscape dog, (B) single human and (C) paired wild animals. The circles represent fixations and the lines saccades, together they show how the dog’s gaze travelled across the image. The second row illustrates the head and body areas of interest, which together constitute the object area, of the same images: (D) landscape dog AOIs, (E) single human AOIs and (F) paired wild animals AOIs. Images A & D are from 123RF, images B & E are from Adobe stock and images C & F are from Shutterstock databases.

Altogether, 36 different stimulus images were presented in this study. The stimuli were color photographs of dogs, humans, and wild animals (mammals, e.g., elephants, tigers, pandas). Three categories of images were used: (1) landscape images containing a human or an animal, (2) full body images of a single human or an animal, (3) full body images of two paired humans or animals (12 images per each category, see examples in Fig. 1). Each image category (landscape, single, paired) contained images of dogs, humans, and wild animals (four images of each subtype in all categories). For example, the landscape image category consisted of images of dogs, humans, and wild animals within a landscape. The physical dimensions of the images were (19.2 × 14.6 cm (height × width). The pixel resolution of the images was 725 × 550 pixels, overlaid on a grey background of 1,680 × 1,050 pixels. Stimuli were obtained from 123RF, Shutterstock, Adobe stock, Wikimedia commons, Microsoft clipart and Bigstock databases.

### Experimental procedure

In the test room, the dog was unleashed and allowed to settle at the pre-trained position. During the experiment, the experimenter and the dog’s owner remained behind an opaque barrier, out of sight of the dog. The dog’s behavior was monitored with a webcam (Labtec Webcam 2200), which was on top of the monitor. Experiment center version. 3.0™ software (SensoMotoric Instruments GmbH, Germany) was used to show the images on a 22 inch (47.4 × 29.7 cm) LCD screen. The images were shown on two separate days (the same 36 images were repeated) in a pseudorandomized order. The stimuli were presented in six blocks of seven to nine images (a total of 36 images per dog per day), 3 s per stimulus and with a 500 ms inter-stimulus-interval. The dog was rewarded with a treat after every image block. Thereafter, the dog was free to return to the pre-trained position.

### Data processing

Successful eye gaze recordings were obtained from all 24 dogs, on average 65 ±  2 images per dog. In total, 128 images were excluded from the analysis owing to eye-tracker software problems, the dog lift its head from the chin rest or the dog left its’ place. BeGaze v. 2.4™ software (SensoMotoric Instruments GmbH, Germany) was used to analyze the raw eye movement data. The fixation was scored with a low-speed event detection algorithm calculating potential fixations with a moving window spanning consecutive data points. To be coded as fixation, the minimum fixation duration was 75 ms and the maximum dispersion value *D* = 250 px {D=[max(x) − min(x)] + [max(y) − min(y)]}. Elsewise, the measured sample was defined as part of a saccade. Binocular gaze data was averaged before analyzes.

### Statistical analyses

Each stimulus image was divided into four areas of interest (AOI): (1) the object area, comprising the heads and bodies of the animals or humans depicted in the images (2) the background area comprising the whole image but not the object area (3) the head area comprising the face and neck areas, and (4) the body area comprising the torso, arms and legs (AOI are illustrated in Fig. 1).

Total gaze times (sum of durations of all fixations and saccades) were calculated from the raw binocular data for each AOI (object, background, head and body). Because the sizes of the AOI varied between image categories and species represented in the images, the gaze time was measured as a normalized score (applied from Dahl et al., 2009; Guo, Tunnicliffe & Roebuck, 2010; Somppi et al., 2016), henceforth referred to as “proportional gazing time”. The score was calculated by subtracting the relative AOI size (e.g., the size of the head divided by the size of the whole object) from the relative gaze time (e.g., the total gaze time of the head divided by the total gaze time of the whole object area). Positive gaze score means that the AOI is looked at longer than would be expected according to the size of the area, and negative scores indicate the opposite. The statistical analyses were conducted using SPSS Statistics version 24.0 (IBM, NY, USA). The total gaze time of the head, body, object and background area in family and kennel dogs were compared with repeated-measures analysis of variance (ANOVA) with a between-subjects factor ‘*group*’ (family, kennel) and within-subjects factors ‘*image category’* (landscape, single, paired), ‘*species*’ (dog, human, wild animal) and ‘*AOI area*’ (head, body, object, background). Paired samples t-tests were conducted to clarify the ANOVA results. All results are reported as mean (± standard error of the mean) of proportional gazing times.

## Results

The total gaze time did not differ between family and kennel dogs (between-subjects factor *group*, *F*_1,22_ = 0.024, *p* = 0.877). Instead, main effect of *AOI area* (*F*_2,38_ = 38.9, *p* = 0.001) and interaction effects between *AOI area × group* (*F*_2,38_ = 4.6, *p* = 0.020), *AOI area × species* (*F*_3,62_ = 2.9, *p* = 0.046), *AOI area × image category* (*F*_3,69_ = 10.1, *p* = 0.001) and *AOI area × image category x group* (*F*_3,69_ = 3.2, *p* = 0.028) were found.

**Figure 2:**
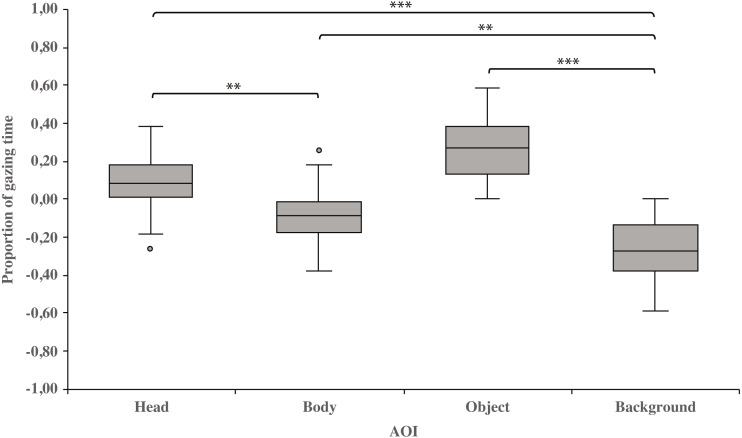
Gazing times of head, body, object and background areas of the images in both dog groups. The mean differences in proportional gazing times of head, body, object and background areas of the images in both dog groups. The mean is shown by a line inside the box and whiskers show the minimum and maximum values of the data. Proportional AOI area gazing times were calculated by subtracting the relative AOI size (e.g., the size of the object divided by the size of the whole image) from the relative gaze time (the total gaze time of the object divided by the total gaze time of the whole image area). Statistically significant differences are represented by asterisks (^∗∗∗^*p* < 0.001 and ^∗∗^*p* < 0.01).

As a main effect, both dog groups gazed at the head area longer than the body (0.10 ± 0.03 and−0.10 ± 0.03, respectively; t_23_ = 3.3, *p* = 0.003) or background area (0.10 ±  0.03 and −0.26 ± 0.03, respectively; t_23_ = 8.6, *p* = 0.001). Furthermore, the body area was gazed longer than the background area (−0.10 ± 0.03 and −0.26 ± 0.03, respectively; t_23_ = 3.4, *p* = 0.002). In addition, the object area was gazed longer than the background area (0.27 ± 0.03 and −0.26 ± 0.03, respectively; t_23_ = 8.3, *p* = 0.001; Fig. 2).

**Figure 3:**
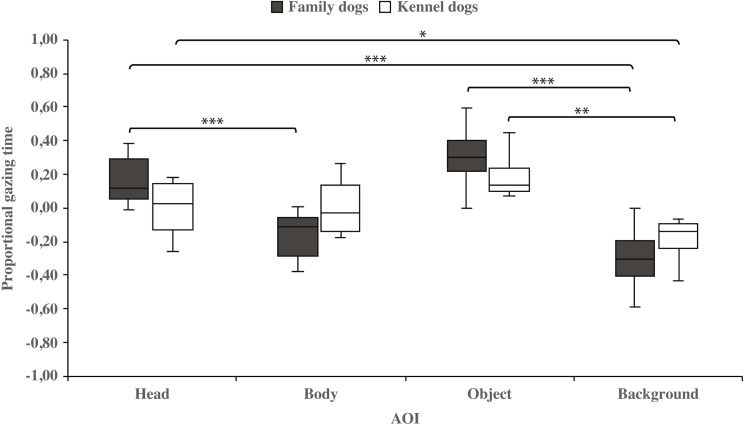
Gazing times of head, body, object and background areas of the images in family dogs and kennel dogs. The mean differences in proportional gazing times of head, body, object and background areas of the images in family dogs (*n* = 16) and kennel dogs (*n* = 8). Statistically significant differences are represented by asterisks (^∗∗∗^*p* < 0.001, ^∗∗^*p* < 0.01 and ^∗^*p* < 0.05).

Within-groups comparisons showed that family dogs gazed at the head area longer than the body (0.15 ±  0.03 and −0.15 ±  0.03, respectively; t_15_ = 4.6, *p* = 0.001) or background (0.15 ±  0.03 and −0.31 ± 0.04, respectively; t_15_ = 11.0, *p* = 0.001) areas. In addition, gazing time was longer for the object area than for the background (0.31 ± 0.04 and −0.31 ± 0.04, respectively; t_15_ = 8.0, *p* = 0.001) areas in family dogs. Furthermore, the body area gazing time was longer than the background area gazing time (−0.15 ± 0.03 and −0.31 ± 0.04, respectively; t_15_ = 2.7, *p* = 0.018). Also in kennel dogs, the gazing time was longer for the head than the background (0.01 ± 0.05 and −0.18 ± 0.04, respectively; t_7_ = 3.3, *p* = 0.012) area. Furthermore, kennel dogs gazed at the object area longer than the background area (0.18 ±  0.04 and −0.18 ±  0.04, t_7_ = 4.1, *p* = 0.005; Fig. 3 & Table 1).

**Table 1:** AOI area gazing times in family and kennel dogs. Paired samples *t*-test results of AOI area gazing times in family and kennel dogs.

AOI	Dog group	Proportional gaze time mean (SEM)	AOI		AOI		AOI	
			Body		Object		Background	
			*t*	*p*	*t*	*p*	*t*	*p*
Head	Family	0.15 (0.03)	4.6	0.001^*^	–	–	11.0	0.001^*^
	Kennel	0.01 (0.05)	0.1	0.930	–	–	3.3	0.012^*^
Body	Family	−0.15 (0.03)	–	–	–	–	2.7	0.018^*^
	Kennel	−0.01 (0.05)	–	–	–	–	2.1	0.075
Object	Family	0.31 (0.04)	–	–	–	–	8.0	0.001^*^
	Kennel	0.18 (0.04)	–	–	–	–	4.1	0.005^*^
Background	Family	−0.31 (0.04)	2.7	0.018^*^	8.0	0.001^*^	–	–
	Kennel	−0.18 (0.04)	2.1	0.075	4.1	0.005^*^	–	–

**Notes.**

* Significant pairwise comparison (*p* < 0.05).

**Figure 4:**
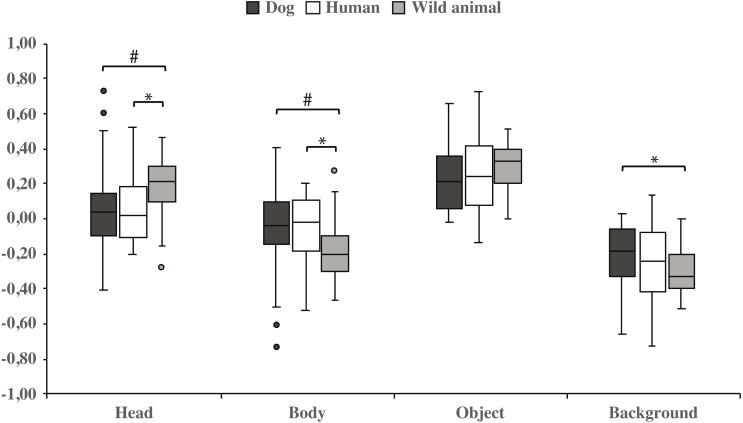
Gazing times of head, body, object and background areas of different species in both dog groups. The mean differences in proportional gazing times of head, body, object and background areas of different species in both dog groups. Statistically significant differences are represented by asterisk (^∗^*p* < 0.05) and statistical trends are marked by (^#^*p* = 0.05).

Both dog groups gazed longer at the head area in wild animal images *vs*. dog images (0.18 ± 0.04 and 0.06 ± 0.06, respectively; t_23_ = −2.1, *p* = 0.050, statistical trend) and likewise longer in wild animal *vs.* human images (0.18 ± 0.04 and 0.07 ±  0.04, respectively; t_23_ = −2.1, *p* = 0.043). The body area was gazed longer in images containing dogs *vs.* wild animals (−0.06 ±  0.06 and −0.18 ±  0.04, respectively; t_23_ = 2.1, *p* = 0.050, statistical trend), and also in images containing humans *vs.* wild animals (−0.07 ± 0.04 and −0.18 ± 0.04, respectively; t_23_ = 2.1, *p* = 0.043). In addition, the background was gazed longer in images containing dogs *vs.* wild animals (−0.22 ± 0.04 and −0.31 ±  0.03, respectively; t_23_ = − 2.1, *p* = 0.048; Fig. 4 & Table 2).

**Table 2:** Different species AOI area gazing times in both dog groups. Paired samples *t*-test results of different species AOI area gazing times in both dog groups.

AOI	Proportional gaze time mean (SEM)		*t*	*p*	Proportional gaze time mean (SEM)		*t*	*p*	Proportional gaze time mean (SEM)		*t*	*p*
	Dog	Human			Dog	Wild animal			Human	Wild animal		
Head	0.06 (0.06)	0.07 (0.04)	−0.1	0.952	0.06 (0.06)	0.18 (0.04)	−2.1	0.050^#^	0.07 (0.04)	0.18 (0.04)	−2.1	0.043^*^
Body	−0.06 (0.06)	−0.07 (0.04)	0.1	0.952	−0.06 (0.06)	−0.18 (0.04)	2.1	0.050^#^	−0.07 (0.04)	−0.18 (0.04)	2.1	0.043^*^
Object	0.23 (0.04)	0.26 (0.05)	−0.7	0.495	0.23 (0.04)	0.31 (0.03)	−1.9	0.069	0.26 (0.05)	0.31 (0.03)	−1.3	0.217
Background	−0.22 (0.04)	−0.26 (0.05)	1.0	0.340	−0.22 (0.04)	−0.31 (0.03)	−2.1	0.048^*^	−0.26 (0.05)	−0.31 (0.03)	1.3	0.215

**Notes.**

* Significant pairwise comparison (*p* < 0.05).

# Statistical trend (*p* = 0.05).

**Figure 5:**
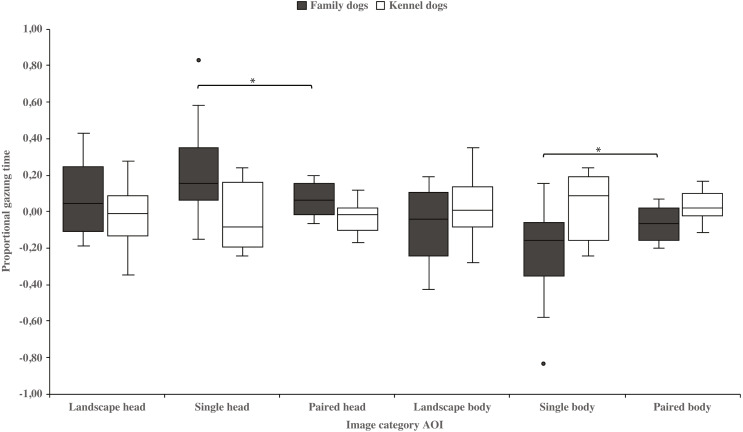
Gazing times of head and body areas between image categories in family and kennel dogs. The mean differences in proportional gazing times of head and body areas between image categories in family and kennel dogs. Statistically significant differences are represented by asterisks (^∗^*p* < 0.05).

**Figure 6:**
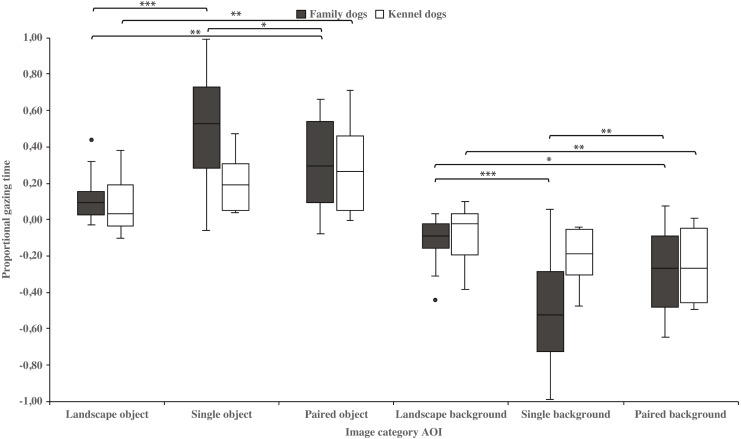
Gazing times of object and background areas between image categories in family and kennel dogs. The mean differences in proportional gazing times of object and background areas between image categories in family and kennel dogs. Statistically significant differences are represented by asterisks (^∗∗∗^*p* < 0.001, ^∗∗^*p* < 0.01 and ^∗^*p* < 0.05).

Family dogs gazed longer at the head area in images containing a single *vs.* paired human or animal images (0.22 ± 0.06 and 0.07 ± 0.02, respectively; t_15_ = 2.7, *p* = 0.017). Family dogs also gazed longer at the body area in paired *vs.* single human or animal images (−0.07 ± 0.02 and −0.22 ± 0.06, respectively; t_15_ = −2.7, *p* = 0.017; Fig. 5). Furthermore, they gazed longer at objects in single human or animal *vs.* landscape images (0.49 ±  0.08 and 0.11 ±  0.03, respectively; t_15_ = −4.9, *p* = 0.001) and likewise longer in paired human or animal *vs.* landscape images (0.31 ± 0.06 and 0.11 ±  0.03, respectively; t_15_ = −3.2, *p* = 0.007) and also longer in single human or animal *vs.* paired images (0.49 ±  0.08 and 0.31 ± 0.06, respectively; t_15_ = 2.4, *p* = 0.030). Instead, family dogs gazed longer at the background area in landscape *vs.* single (−0.11 ± 0.03 and −0.49 ± 0.08, respectively; t_15_ = 4.9, *p* = 0.001) or paired images (−0.11 ±  0.03 and −0.28 ±  0.05, respectively; t_15_ = 3.0, *p* = 0.010). Likewise, they gazed longer at the background areas in paired *vs.* single images (−0.28 ±  0.05 and −0.49 ±  0.08, respectively; t_15_ = −3.5, *p* = 0.003; Fig. 6). For kennel dogs, the gazing times of object area were also longer for paired human or animal *vs.* landscape images (0.29 ±  0.09 and 0.08 ±  0.06, respectively; t_7_ = −4.0, *p* = 0.005). Kennel dogs also gazed longer at the background area in landscape *vs.* paired human or animal images (−0.08 ± 0.06 and –0.26 ±  0.07, respectively; t_7_ = 3.5, *p* = 0.009; Fig. 6 & Table 3).

## Discussion

In this work, we studied dogs’ visual scanning of images of natural scenes containing dogs, humans, and exotic wild animals. The present work was the first canine eye tracking study using stimuli species other than dogs or humans, and using images, which varied in composition (landscapes and close-up images of humans and animals). Generally, dogs identified the living creatures from the images and focused more attention on their heads relative to the background, which is very much in line with the previous eye-tracking studies (e.g., Téglás et al., 2012; Somppi et al., 2012; Barber et al., 2016). Human and non-human primates are known to concentrate their gaze on the human and animal figures with particular attention paid to the faces of those figures (e.g., Yarbus, 1967; Henderson & Hollingworth, 1999; Kano & Tomonaga, 2009; Machado et al., 2011). Furthermore, human and non-human primates are able to detect living creatures from a landscape image quickly and accurately, even if they are partially hidden and relatively small compared to the background (Fabre-Thorpe, Richard & Thorpe, 1998; Thorpe et al., 2001; New, Cosmides & Tooby, 2007). Our current results suggest a similar, spontaneous detection of living creatures by dogs. The dogs’ preference for biologically relevant information within visual scenes is consistent with the “life detector mechanism”; a basic visual method for focusing attention towards and detecting other animals (reviewed in Rosa-Salva, Mayer & Vallortigara, 2015). Dogs’ preference for biological information is also highlighted in the recent studies using point-light animations of biological motion (Kovács et al., 2016; Ishikawa et al., 2018). In addition to the whole biological creatures, dogs generally fixated more on the heads of animals and humans rather than backgrounds of the images, which further emphasizes the importance of faces in social animals’ visual processing (for a review, Leopold & Rhodes, 2010).

**Table 3:** Different image categories AOI area gazing times in family and kennel dogs. Paired samples *t*-test results of different image categories AOI area gazing times in family and kennel dogs.

AOI	Dog group	Proportional gaze time mean (SEM)		*t*	*p*	Proportional gaze time mean (SEM)		*t*	*p*	Proportional gaze time mean (SEM)		*t*	*p*
		Image category				Image category				Image category			
		Landscape	Single			Landscape	Paired			Single	Paired		
Head	Family	0.08 (0.05)	0.22 (0.06)	−1.9	0.084	0.08 (0.05)	0.07 (0.02)	0.1	0.947	0.22 (0.06)	0.07 (0.02)	2.7	0.017^*^
	Kennel	−0.02 (0.07)	−0.05 (0.07)	0.5	0.600	−0.02 (0.07)	−0.03 (0.03)	0.2	0.832	−0.05 (0.07)	−0.03 (0.03)	−0.3	0.809
Body	Family	−0.08 (0.05)	−0.22 (0.06)	1.9	0.084	−0.08 (0.05)	−0.07 (0.02)	−0.1	0.947	−0.22 (0.06)	−0.07 (0.02)	−2.7	0.017^*^
	Kennel	0.02 (0.07)	0.05 (0.07)	−0.5	0.600	0.02 (0.07)	0.03 (0.03)	−0.2	0.832	0.05 (0.07)	0.03 (0.03)	0.3	0.809
Object	Family	0.11 (0.03)	0.49 (0.08)	−4.9	0.001^*^	0.11 (0.03)	0.31 (0.06)	−3.2	0.007^*^	0.49 (0.08)	0.31 (0.06)	2.4	0.030^*^
	Kennel	0.08 (0.06)	0.20 (0.05)	−2.1	0.074	0.08 (0.06)	0.29 (0.09)	−4.0	0.005^*^	0.20 (0.05)	0.29 (0.09)	−1.0	0.341
Background	Family	−0.11 (0.03)	−0.49 (0.08)	4.9	0.001^*^	−0.11 (0.03)	−0.28 (0.05)	3.0	0.010^*^	−0.49 (0.08)	−0.28 (0.05)	−3.5	0.003^*^
	Kennel	−0.08 (0.06)	−0.20 (0.05)	2.1	0.069	−0.08 (0.06)	−0.26 (0.07)	3.5	0.009^*^	−0.20 (0.05)	−0.26 (0.07)	0.7	0.495

**Notes.**

* Significant pairwise comparison (*p* < 0.05).

Contrary to our expectations, dogs’ gazing behavior was not greatly altered by the species depicted in the images. Dogs gazed at the head areas in wild animal images longer than in human or dog images. Possibly, the naturally novel wild animals attracted dogs’ attention to a higher degree than humans or dogs (Fantz, 1964), and this kind of natural novelty might reflect the general visual experience of the observer. Previously, rhesus monkeys showed an increased pupillary response, when they viewed videos containing non-primate animals (e.g., marine mammals) and landscapes (e.g., polar), for which they have had limited exposure during their lives (Machado et al., 2011). The ability to detect and respond quickly to novel events is essential for survival in the nature (Ranganath & Rainer, 2003), which is also highlighted in our current results. In previous studies using facial images, dogs preferred to look at conspecific faces over those of humans (e.g., Somppi et al., 2012; Somppi et al., 2014; Bognár, Iotchev & Kubinyi, 2018). However, when they viewed photographs presenting whole bodies within a scene, they have gazed more at humans than dogs (Törnqvist et al., 2015). This difference means that factors other than merely the species influences viewing behavior (Dahl, Logothetis & Hoffman, 2007). For example, human infants show spontaneous gazing preferences toward dog versus human pictures (Gergely et al., 2019), probably owing to the novelty of the stimuli. In our study, no great differences were found between the species depicted, possibly because the images were emotionally neutral full-body figures with scant social gestures and three different types of images were used. In rhesus macaques, gaze distribution in viewing unfamiliar non-conspecific animals varied systematically according to their phylogenetic distance from the viewed species. It was also dependent upon the social and emotional contexts of the image (McFarland et al., 2013).

The present study showed, for the first time, that the image composition affects the dogs’ gazing behavior. In general, dogs spent less time gazing at the object areas in landscape images, where the size of the target was small relative to the image size, which contrasts from the other image types. From this result we infer that dogs may have had difficulty in locating objects within the landscape images. The visual acuity and the ability to detect brightness of the canine visual system is lower than in humans (Pongrácz et al., 2017; for a review, Byosiere et al., 2018). Therefore, the size of stimuli that dogs can see clearly may vary greatly (e.g., review in Byosiere et al., 2018; Tanaka et al., 2000; Lind et al., 2017). In this study, dogs were shown color images, which may also have affected their ability to perceive these images. Dogs have dichromatic color vision, but the data determining which colors dogs can discriminate are controversial (e.g., Neitz, Geist & Jacobs, 1989; Miller & Murphy, 1995; for a review, Byosiere et al., 2018). Recent studies suggest that dogs are unable to distinguish red from green color and that color information might be more important than brightness when dogs are required to choose between stimuli (Kasparson, Badridze & Maximov, 2013; Siniscalchi et al., 2017).

Additionally, family dogs gazed at the head areas of single animals or humans longer than images of paired animals or humans. Conversely, family dogs gazed at the body areas of animals or humans longer in the paired rather than the single image conditions. Thus, family dogs’ gazing preference for the head area was clearly shown in images containing single animals, likely indicating that fixations may have been spread more widely where there were two head areas i.e., in the paired animal images. In these images, body areas may have drawn the attention of family dogs’, because of the social bodily gestures that are present in these images. In the paired images, two animals or humans were standing or sitting close to each other. Interestingly, kennel dogs’ gazing behavior did not differ between head and body areas in the single or paired images. This result is in line with our previous studies, where kennel dogs gazed at scenes of social interaction and facial images less than family dogs (Törnqvist et al., 2015; Somppi et al., 2014). Since family dogs have had more opportunities for social interactions than kennel dogs, they may be more interested and have a greater ability to search whether there are social gestures presented in those images. However, the differences between family and kennel dogs’ gazing behavior may be more evident with socially richer stimuli than the ones used in the current study.

Other than the latter difference between the two study groups, family and kennel dogs gazed at the images in a similar manner, focusing their gaze on the head and object areas of the images. This result suggests that the basic viewing processes are the same regardless of the social living environment and life experiences of the participants. Several previous studies have shown that humans and some other newborn vertebrates have visual predispositions for the head region of living creatures (e.g., Johnson et al., 1991; for a review, Morton & Johnson, 1991). Monkey infants and newly hatched chicks show visual preference for faces, even when there has been no previous exposure to faces (Sugita, 2008; Rosa-Salva et al., 2011). Despite their more restricted social living environment, kennel dogs had daily contact with humans and other beagle dogs. This might have diminished the differences in gazing behavior between family and kennel dogs. In addition, the study populations were not equal in number between family and kennel dogs (2:1), which may have affected our results.

In this study, dogs were trained to remain still during image presentation, because spontaneous movement during the eye tracking recordings can cause serious artifacts and loss of data. However, dogs were not trained to target their gaze at the images or screen. During the experiments, dogs were rewarded with a treat after every image block, which was considered necessary in order to prevent frustration and to ensure that dogs were motivated. It is unlikely that the dogs learnt to associate rewards with certain presented images or that a particular viewing strategy would have been reinforced. Dogs were rewarded only when the screen was blank, and the last image of the block was always different making it hard to learn any specific gazing strategy.

## Conclusions

In summary, when viewing photographs of natural scenes, dogs focus their gaze on the informative areas such as the bodies and heads of living creatures, as previously shown for primates. Gazing behavior was dependent on the size and species of the target animals in the images for both dogs living in a socially rich and stimulating family environment and dogs living in a more restricted kennel environment. Nevertheless, there were minor differences in viewing behavior of these dog groups, suggesting that social experiences modulate dogs’ spontaneous viewing patterns when looking at natural scenes.

##  Supplemental Information

10.7717/peerj.10341/supp-1 Supplemental Information 1 Dog eye gaze data Family and kennel dogs’ eye gaze data (ms) towards the stimulus images’ areas of interest.

## Additional Information and Declarations

### Data Availability

The following information was supplied regarding data availability: The raw eye tracking data is available in the Supplemental File.
